# GOAL: A software tool for assessing biological significance of genes groups

**DOI:** 10.1186/1471-2105-11-229

**Published:** 2010-05-06

**Authors:** Alain B Tchagang, Alexander Gawronski, Hugo Bérubé, Sieu Phan, Fazel Famili, Youlian Pan

**Affiliations:** 1 Knowledge Discovery Group, Institute for Information Technology, National Research Council, Canada, 1200 Montreal Road, Ottawa, ON K1A 0R6 Canada

## Abstract

**Background:**

Modern high throughput experimental techniques such as DNA microarrays often result in large lists of genes. Computational biology tools such as clustering are then used to group together genes based on their similarity in expression profiles. Genes in each group are probably functionally related. The functional relevance among the genes in each group is usually characterized by utilizing available biological knowledge in public databases such as Gene Ontology (GO), KEGG pathways, association between a transcription factor (TF) and its target genes, and/or gene networks.

**Results:**

We developed *GOAL*: **G**ene **O**ntology **A**na**L**yzer, a software tool specifically designed for the functional evaluation of gene groups. *GOAL *implements and supports efficient and statistically rigorous functional interpretations of gene groups through its integration with available GO, TF-gene association data, and association with KEGG pathways. In order to facilitate more specific functional characterization of a gene group, we implement three GO-tree search strategies rather than one as in most existing GO analysis tools. Furthermore, *GOAL *offers flexibility in deployment. It can be used as a standalone tool, a plug-in to other computational biology tools, or a web server application.

**Conclusion:**

We developed a functional evaluation software tool, *GOAL*, to perform functional characterization of a gene group. *GOAL *offers three GO-tree search strategies and combines its strength in function integration, portability and visualization, and its flexibility in deployment. Furthermore, *GOAL *can be used to evaluate and compare gene groups as the output from computational biology tools such as clustering algorithms.

## Background

In the midst of the functional genomic era, DNA microarray technology is widely used to study the expression level of thousands of genes under different experimental conditions or time points. Grouping of genes is a norm in functional genomics. Statistical criteria and biological hypotheses, combined with well defined computational techniques such as clustering [1-5], are then used to group genes that could be functionally related. Post clustering processes, such as functional characterization that relate the gene expression profile with the functional significance must be conducted. A prevailing procedure in functional characterization is to search the annotation of genes in respective group through Gene Ontology (GO) [6-8], KEGG pathways [9], REACTOME pathways [10], transcription factor (TF) gene association data [11], and/or gene networks. These post processing steps allow identifying biological functions which are highly represented in the given group of genes.

The Gene Ontology provides controlled vocabularies for the description of the molecular function, biological process, and cellular component of gene products [6-8]. TF-gene association data describes with a given probability whether or not a given gene is regulated by a specific transcription factor. One of the goals of identifying clusters of functionally related genes is to associate the genes and their interconnections with known biological pathways. In this regard, the KEGG pathways database provides a very rich resource to test this hypothesis. Its aim is to link individual level information such as genes, proteins, enzymes, with system level information such as interactions, enzymatic reactions, and pathways.

There are several packages available for functional characterization of gene groups: FuncAssociate [12], GOstat [13], BINGO [14], DAVID [15,16], CLENCH [17], FATIGO [18], GOSt [19], US FDA's ArrayTrack [20], EXPANDER [21], and many others that are referenced in the microarray tools' section of the GO website [22-24]. However, each of these has its limitations. Among these limitations has been the failure to consider which genes were actually assayed in the experiment that produced the list of interesting genes [12]. Several of these pioneering functional evaluation tools have mainly been implemented using only the GO annotations [12-14]. Some of these tools, such as BINGO [14] and CLENCH [17], are available as a part of some broader software packages or dedicated to specific applications, and do not offer flexibility in terms of deployment and biological applications. BiNGO [14] only offers GO evaluation and it is designed as a plug-in to Cytoscape [25]. CLENCH [17] is specifically designed to allow *Arabidopsis thaliana *researchers to perform automated retrieval of GO annotations from TAIR database [26]. DAVID [15,16], FATIGO [18], GOSt [19], ARRAYTRACKTM [20], and EXPANDER [21] offer the use of more advanced functions, such as protein-protein interactions and/or KEGG pathways, than the others. However, DAVID does not offer the possibility for user to incorporate available TF-gene association data in their analysis. Although FATIGO and EXPANDER make use of TF binding data, the user is limited to use the one provided by the software. Furthermore, packages such as FATIGO and several others that are only available as web server application make the users completely dependent on the availability of the server and unable to update associated data, such as GO data files.

In this paper we introduce a **G**ene **O**ntology **A**na**L**yzer, *GOAL*, which is a software tool specifically designed for the biological evaluation of groups of genes. *GOAL *implements and supports efficient and statistically rigorous biological interpretations of groups of genes through its integration with the Gene Ontology, available TF-gene association data, and KEGG pathways. Unlike other GO analysis tools, *GOAL *provides three GO-tree search strategies to detect the GO terms that are specific to a gene group and enables users to update source data, such as GO files, at anytime. Additionally, *GOAL *offers flexibility in terms of integration and deployment.

The hypotheses behind the use of Gene Ontology, TF-gene associations, the KEGG pathways in the implementation of *GOAL *are described as follows. Ideally, a good clustering algorithm identifies genes with similar expression patterns, which are probably co-regulated by the same transcription factors [1-5]. In addition, genes that are co-expressed frequently participate in the same or related biological pathways [1-5]. In both cases, if the genes in co-expressed groups are functionally related, they should be enriched with and annotated to related GO terms, involved in related biological pathways and regulated by related transcription factors [3].

The rest of this paper is described as follows. First, we describe the implementation. Then we illustrate the use of *GOAL *through application examples, and show how the *GOAL *package can be deployed. Finally, we discuss and conclude.

## Implementation

*GOAL *is a Java module designed specifically to provide the functional characterization of groups of genes. Within the input list of genes, *GOAL *identifies statistically significant GO terms, TF-gene associations, and KEGG pathways. The framework relies on the *p*-value computed using a *Fisher's exact test *on a *2 *× *2 *contingency table derived either from the GO enrichment level, TF-gene associations, or KEGG pathways. The output of *GOAL *is a list of significant GO terms, TF-gene associations or KEGG pathways together with their *p-values *that state how significant the associations are for the list of input genes.

### Statistical Significance

For a given set of input genes (gene id types are provided in the user manual) and a reference gene list, *GOAL *determines all annotated GO terms and their parental GO terms (defined by the input parameter *max path length from leaf *or *min path length from root*), all TF-gene associations, and all associations with KEGG pathway for the set of input genes. It then counts the number of appearances of each GO term, each TF-gene association, and each association with a KEGG pathway for the genes within the group and for the reference genes. *Fisher's exact test *is performed to determine whether or not the observed difference is significant. This will result in a *p-value *for each GO term, TF-gene association, or the association with a KEGG pathway that the observed counts could have been achieved from the background list by chance. The *p-value *is evaluated using the hypergeometric distribution for each GO term, TF-gene association, or KEGG pathway:(1)

where *n *is the number of genes in the intersection between the input gene list and those involved in the GO term, TF-gene association, or KEGG pathway of interest; *N *is the total number of unique genes in the reference gene list, *K *is the total number of genes that are annotated to the GO term, TF-gene association, or KEGG pathway of interest, and *I *is the number of genes in the input dataset. To give more statistical power to *GOAL*, the computed *p-value *is further corrected using one of the three multiple-testing corrections (Bonferroni correction, Bonferroni step-down, and Benjamini false discovery rate) [27]. These multiple-testing correction approaches are listed in the order of their stringency, with the Bonferroni being the most stringent, and the Benjamini FDR being the least stringent. The more stringent a multiple-testing correction, the less false positive observations are allowed.

### Gene Ontology Search Strategies

Three GO-tree search strategies are implemented in *GOAL*: I) *max path length from leaf*, II) *min path length from root*, and III) the combination of I and II which we call *combined search strategy *in this article. Note that Strategies I and III are specific in *GOAL*, whereas, Strategy II is used in most other Gene Ontology tools mentioned above.

#### Max path length from leaf

here, we define *leaf *as the most specific GO term of a gene. This GO term may not be a leaf node along the GO-tree hierarchy. But there is no GO-term at the current stage that is more specific in defining the gene. *GOAL *first locates the most specific GO term of the gene along the hierarchy (leaf), users are allowed to define the maximum number of steps along its parental path to backtrack to a parent, grand-parent or great grand-parent, etc., of the GO term. This parameter defines the GO-tree search space to the *k*^*th *^parental GO term from the most specific GO term. For example, if a user wants to search the most specific GO term only, the value is 0.

#### Min path length from root

*GOAL *first locates the most general GO term of the gene along the hierarchy (root, e.g. Biological Process); users are allowed to define a threshold number of steps from the root along the offspring path. This threshold number of steps is the cut off level in the GO-tree. Most existing tools implement only this strategy.

#### Combined search

This is a combination of Strategies I and II. When this approach is used, *GOAL *first locates the most specific GO term as described in strategy I. Then, the *p-value *of each one of them is computed as in strategy II. By doing this, *GOAL *considers the most specific GO terms, while discarding the ones that are more general than the threshold set by Strategy II. Another advantage is that it also considers genes that are associated with parental GO terms of the most specific GO terms located within strategy II.

### Development

*GOAL *is implemented entirely in Java. It is available as an executable jar file and technically works with all operating system supporting Java SDK 1.6 or later. We tested under Microsoft Windows (XP, Vista) and Linux systems (Ubuntu 9.10) and provided a start-up script for these two operating systems. The GUI is written using the Java Swing and AWT libraries which are part of the Java SDK. For network communication between clients and the server, the Java RMI library is used, which is also part of the SDK. Other packages included are Jargs (command line interface) and CSVreader (File IO). *GOAL *also makes use of external Gene Ontology and gene annotation files [6-8]. The GO files are accessible for analysis by *GOAL *using a partial, modified version of the BioJava 1.6.1 API. *GOAL *can download these files directly from the Gene Ontology [22] or European Bioinformatics Institutes [28]. All GO file can be updated at any time by clicking an update button.

**Figure 1:**
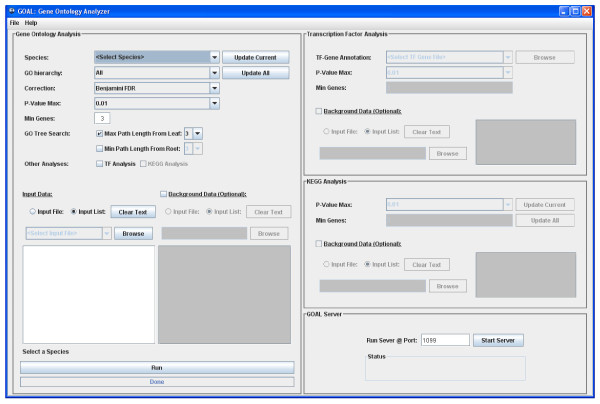
***GOAL *input interface**. The GUI is divided into four sections: GO analysis, TF-gene analysis, KEGG pathways analysis, and the *GOA*L server. The first three sections are further divided into subsections, where the user can specify the input parameters, the list of gene identifiers, which specify the group of genes of interest and the background data. Users have an option to input their own list of reference. The executable button is located at left bottom of the interface.

The GUI provides user convenience of inputting the list of genes and selecting various parameters. Users first specify a list of gene identifiers of interest in the text area of the input interface (Figure. 1). The list of gene identifiers can also be uploaded as a delimited data file specified in the User Manual (accessible through the help menu of *GOAL*). It is the same for reference gene list if users desire to input their own references. By default, the system provides the whole genome of the selected species as the reference. Next, the user specifies the gene annotation source (species) from a drop-down list, which specifies the GO file that *GOAL *will use to seek for annotation and to calculate the *p*-values. A list of default parameters (GO hierarchy, *p*-value correction, maximum *p*-value, maximum path length from leaf and/or minimum path length from root, minimum number of genes in a term) is also available in the GUI (Figure 1). The user may change these default values if they desire. If the TF-gene association data for the corresponding study and species are available, users can upload them as tab delimited data files. Finally, users can also choose the KEGG pathway evaluation if the selected species is supported.

The TF-gene association file is compiled by the user. As we mentioned earlier, the TF-gene associations data can be derived from Chromatin Immunoprecipitation (ChIP) [11] experiments. They can also be compiled from literature and/or TF databases of well known and well characterized biological interactions such as TRANSFAC [29], JASPAR [30], and RegulonDB [31], etc. For example, TRANSFAC is a database on eukaryotic transcriptional regulation. The database contains data on transcription factors, their target genes and their experimentally-proven binding motifs in genes' promoter. The TF-gene association data is represented as a matrix, where the rows correspond to genes and the columns to transcription factors. The entries of the matrix are either 0 or 1, with 1 for a known interaction between a TF and its target gene and 0 for no interaction.

**Figure 2:**
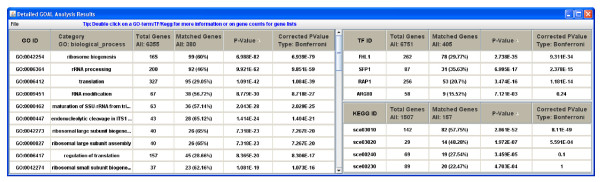
***GOAL *output of Gene Ontology, transcription factor and target gene association, and KEGG metabolic pathways gene association evaluation of the *Saccharomyces cerevisiae *amino acid starvation data**.

**Figure 3:**
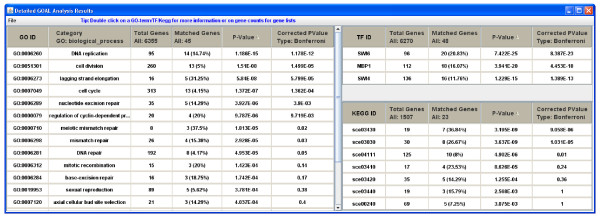
***GOAL *output of Gene Ontology, transcription factor and target gene association, and KEGG metabolic pathways gene association evaluation of the *Saccharomyces cerevisiae *cell cycle data**.

**Figure 4:**
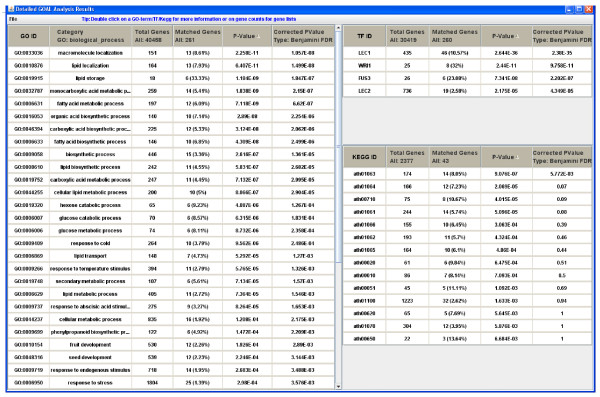
***GOAL *output of Gene Ontology, transcription factor and target gene association, and KEGG metabolic pathways gene association evaluation of the *Brassica napus *data**.

Following the input phase, the *GOAL *algorithm executes as described above, and new windows will appear displaying the functional evaluation results. Few examples are shown in Figures 2, 3 and 4. From these new windows, a user has the option to explore or save the results as a comma separated values (CSV) file. Furthermore, the GO terms in the output contain hypertext links to a visualization tool for the GO hierarchy; genes are linked to NCBI [32], and KEGG pathways ids are linked to KEGG database [9].

*GOAL *is available as Additional File 1. A quick video tutorial is also available as Additional File 2. The most recent version of *GOAL *can be downloaded directly from the *GOAL *project website: http://bioinfo.iit.nrc.ca/GOAL/.

## Results

### Application Examples

We used three different datasets to test *GOAL*. Groups of co-expressed genes were obtained from each set of data using a well defined clustering algorithm [3,4]. The first dataset is a group of co-expressed gene from the time-series gene expression data of the *Saccharomyces cerevisiae *amino acid (AA) starvation dataset [33]. For the TF-gene association data, we used the published AA starvation ChIP-chip dataset on 34 transcription factors [34]. Figure 2 shows the combined results of the GO analysis, TF-gene association, and KEGG pathway association of an identified gene group using *GOAL*. The results shown in Figure 2 are consistent with experimental result; the transcription factors FHL1 (p-value = 9.31e-34) and SFP1 (p-value = 1.18e-15) regulate many genes involved in ribosome biosynthesis [35]. This is the most significant GO biological process in this cluster. This is also consistent with the KEGG pathway analysis where the ribosome pathway (sce03010, p-value = 8.11e-49) is the most significant.

The second dataset is a group of co-expressed genes from the *Saccharomyces cerevisiae *cell cycle dataset [36]. The TF-gene association data we used here are derived from [37]. This dataset contains 113 yeast TFs and their target genes. Figure 3 shows the combined results of the GO analysis, TF-gene association, and KEGG pathway association of an identified cluster using *GOAL*. The most significant GO biological processes identified for this cluster is "cell cycle", with a *p*-value = 8.6e-09 (Figure 3). KEGG pathways and TF-gene results are consistent with the GO analysis. TF-gene analysis shows that the transcription factors MBP1 (P-value = 4.4e-18), SWI4 (p-value = 1.3e-13) and SWI6 (p-value = 8.3e-23) have significantly overrepresented number of target genes in this cluster. These three TFs are known to participate in the two major transcription complexes regulating G1/S transition in cell cycle process [38]. KEGG pathways analysis also revealed the cell cycle pathway (sce04111, p-value = 4.6e-03) and DNA replication pathway (sce03030, p-value = 5.1e-06). This indicates that the three parts of analysis are consistent.

The third dataset is a co-expressed gene group from the time series gene expression data of *Brassica napus *during seed development [39]. Since the *B. napus *genome is not yet complete, we use the orthologs in *A. thaliana*, a close relative of *B. napus*, for computational analysis. The search for orthologs in *A. thaliana *is done by blasting the sequences of interest *B. napus *genes against the TAIR [40] database (version 8) and keeping only the matches with scores better than 1e-20. The TF-gene interactions matrix was inferred through a carefully selected literature search, bias towards experimental evidence [41-43]. More precisely, we compiled from the literature a set of *A. thaliana *TFs that have been shown by microarray experiments and/or quantitative RT-PCR to promote, suppress, or induce genes that are related to seed development, FA metabolism and other related biological processes such as lipid metabolism and biosynthetic process, with a *p-value *< 0.01 [36-38]. Figure 4 shows the results of the GO analysis, TF-gene association, and the involvement in the metabolic pathways. GO biological process shows that most of the genes in this group are highly enriched under seed development, lipid and fatty acid biosynthetic and metabolism. This is consistent with the TF-gene association results and KEGG pathway results. This group of genes is significantly co-regulated by the transcription factors LEC1 (p-value = 2.3e-35), LEC2 (P-value = 4.3e-05), FUS3 (p-value = 2.2e-07), and WRI1 (p-value = 9.7e-11). Significant number of genes in this group are involved in the biosynthesis of alkaloids derived from shikimate pathway (ath01063, 14 genes), phenylpropanoid (ath01061, 14 genes), which are related with fatty acid biosynthesis (ath00061) and metabolism (ath00071), and citrate cycle (ath00020). These results are consistent with the fact that the LEC1 and LEC2 have been shown to play major roles during seed development, and also with the fact that LEC1 function is partially dependent on FUS3 and WRI1 in the regulation of FA and FA-derived complex lipid [41].

**Figure 5:**
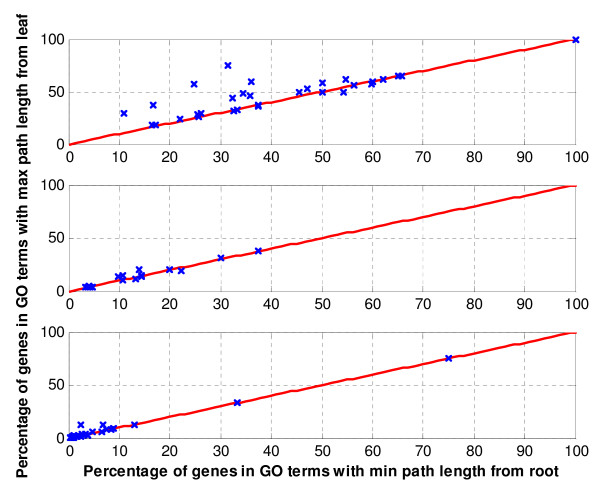
**Comparative analysis of the *max path length from leaf *(Strategy I) and the *min path length from root *(Strategy II) approaches in terms of percentage of genes in each GO category**. Each row represents one of the three datasets: *Saccharomyces cerevisiae *amino acid starvation data, *Saccharomyces cerevisiae *cell cycle data, and *Brassica napus *data respectively. The *y*-axis represents the percentage of gene in a GO terms depicted using Strategy I (0, the most specific term by itself; no parent is considered). Likewise, the x-axis represents the percentages of genes in the same GO terms identified using Strategy II (3, a default in several tools). Points above the central diagonal line represent the GO terms with higher percentage of genes in Strategy I than Strategy II, whereas points below the line, the GO terms with higher percentage of genes identified using Strategy II. As can be seen, Strategy I has the largest number of GO terms with higher percentage of genes.

**Figure 6:**
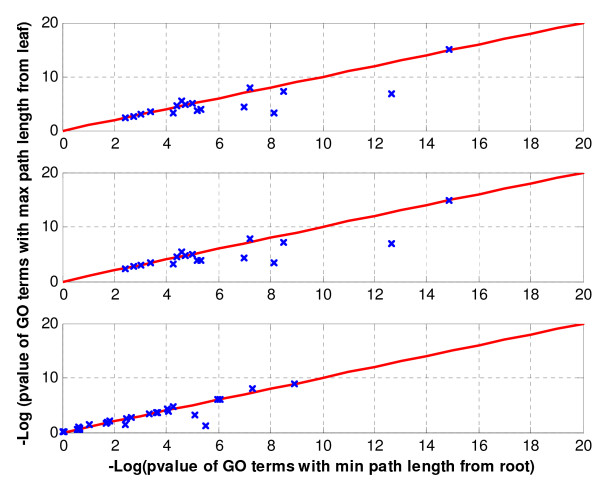
**Comparative analysis of the *max path length from leaf *(Strategy I) and the *min path length from root *(Strategy II) approaches in terms of *p*-value of each GO term**. Each row represents one of the three datasets: *Saccharomyces cerevisiae *amino acid starvation data, *Saccharomyces cerevisiae *cell cycle data, and *Brassica napus *data respectively. The *y*-axis represents the minus log *P*-value for GO enrichment using the Strategy I (0, the most specific term by itself; no parent is considered). The *x*-axis represents the minus log *P*-value for GO enrichment using Strategy II (3, a default in several tools). Points above the central diagonal line represent the most significant GO-terms in Strategy I, whereas points below the line represent categories with most significant terms in Strategy II. As can be seen, Strategy II has slightly larger number of significant GO terms.

Comparative analysis of GO-tree search Strategies I and II (Figures 5 and 6) shows that Strategy I usually outperforms Strategy II in terms of percentage of genes associated to a GO term (Figure 5). Here, the percentage of genes associated to a GO term corresponds to the number of genes in the intersection of a cluster with a GO term divided by the total number of genes in the background that are annotated to that GO term. Nevertheless, the *p-values *of GO terms in Strategy II are in some cases slightly more significant as compared to the ones in Strategy I (Figure 6). Further analysis showed that several GO terms identified using Strategy II are more generic compared to the ones identified by Strategy I which are more specific. Thus groups of genes in Strategy I are biologically more parsimonious than the ones in Strategy II. Note that, there might be some values for which the two GO-tree search strategies yield the same output. Furthermore, from a statistical perspective, a search strategy that is related to more genes is preferable. But, in biological applications, this assertion is biologically relevant if the GO terms depicted by the corresponding search strategy are more specific. In other words, a search strategy that is related to more genes and that depicts more generic GO terms is less biologically relevant compared to a search strategy that depicts more specific GO terms as strategy I.

The data used for these analyses are provides in the *GOAL *tool as default data and can be exploited by the users in their own research. *GOAL *has also been successfully used to characterize the following six human genes (IFNA4 IL12B IL2RB STAT1 STAT2 IRF9) provided to us by one of the reviewers (Additional File 3), to characterize groups of correlated genes in DNA microarray experimental data of *Arabidopsis thaliana *under pathogens attack [44], and several in house (private) DNA microarray datasets of *Brassica napus *during seed development.

### Deployment

*GOAL *can be used as a standalone tool and also offers flexibility to be integrated with other applications. For example, it can be a plug-in to other computational biology tools, or deployed as a web server application.

#### The standalone application

the tool is packaged into a single executable jar file and only requires Java to be installed. A configuration file is automatically generated on the first run and can be modified to suit the user's need. The installer also creates necessary file structure and a shortcut to a batch file to run the tool. GO files are downloaded from GO database [22] on the first run.

#### Plug-in application

*GOAL *can be easily integrated into other tools with minimal coding by the user. The user can create an instance of the *GOALPlugin *class which gives access to all components of *GOAL*. It can be used to retrieve or display graphical user interface (GUI) components for integration into existing GUIs or display in a separate window. The class also allows for running functional components independently of the GUI in a separate or currently used thread. Furthermore the user can execute the jar file through a provided command line interface.

#### Web server application

Another functionality of *GOAL *is the ability to be used remotely through a java Remote Method Invocation (RMI) server. Along with the standard package, a client package is also available. The client can be installed on any remote computer and communicate with the server machine that host a web server. All the computation initiated by various clients is done by the same server. This creates an organized environment where only the server needs to keep annotation files up-to-date. Furthermore only the server would require suitable hardware to run the computations. This way, the server version can also be a middleware to a database that host gene data. More details are available in the User Manual.

## Discussion

The recent proliferation of high-throughput functional genomics methods has brought with it a variety of ways to produce large lists of genes based on well defined statistical assumptions and biological hypotheses. For example, clustering algorithms are used to collect sets of co-expressed genes. Genes in each group that behave similarly in the expression profile across experimental conditions or time points most likely share similar biological functions and processes, genetic pathways, and probably co-regulated. Several solutions have been offered to address these issues. However, each of these has its limitations, of which, the lack of appropriate corrections for multiple hypothesis testing and the failure to consider whether a gene was actually validated in wet lab experiment are the two most obvious ones [12]. The *GOAL *package addresses this issue by implementing several multiple correction approaches and by allowing users to incorporate ChIP-chip data.

Another important aspect of ontological analysis regardless of the tool or statistical method is the choice of the reference gene list against which the list of differentially regulated genes is compared. Inappropriate choice of reference genes may lead to false functional characterization of a gene list. Khatri and Drǎghici [24] pointed out that only the genes represented on the array, although quite incomplete, should be used as reference list instead of the whole genome as it is a common practice. The *GOAL *package addresses this issue by allowing the users to either use the entire genome of the selected species as the reference, or upload their own reference genes.

In addition, GO allows for the annotation of genes at different levels of abstraction due to its directed acyclic graph (DAG) structure. In this particular hierarchical structure, each GO term can have one or more child terms as well as one or more parent terms. For instance, the same gene list is annotated with a more general GO term such as "metabolic process" at a higher level of abstraction, whereas the lowest level provides a more specific ontology term such as "lipid metabolic process." It is important as in the *GOAL *package, to integrate the hierarchical structure of the GO in the analysis since various levels of abstraction usually give different *p*-values. In this regard, *GOAL *first locate the most specific GO term of the gene (we define it as a leaf) along the hierarchy, users are allowed to define the maximum number of steps along parental path to backtrack to a parent or grand-parent of the GO term. *GOAL *also implements a GO-tree search strategy (min path length from root) common to most existing Gene Ontology packages. Additionally, users may want to combine both strategies to do more specific functional characterization of a gene group. *GOAL *provides the users with three options to determine significant function of a gene group and to better address their research problem.

Other than some GO evaluation tool as implemented in several existing packages, *GOAL *takes a step further by also including the association between transcription factors and their target genes, and gene's involvement in various metabolic pathways in its evaluation process. The co-expressed genes identified through clustering processes are probably co-regulated by common transcription factors and frequently participate in the same or related biological pathways [1-5]. Thus, they should be enriched with certain GO terms, related with certain TFs, and associated with certain biological pathways. *GOAL *is not only capable of evaluating whether or not a given group of genes may be involved in the same genetic pathways, but also capable of evaluating whether or not they might be co-regulated at the transcriptional level.

## Conclusion

We have introduced a new software package for functional characterization of a gene list and to evaluate the functional domain of these genes. *GOAL *integrates Gene Ontology with available TF-gene associations and KEGG pathways information. *GOAL *provides users with three different GO-tree search strategies. *GOAL *is highly portable, can be used as a standalone tool, a plug-in to other computational biology tools, or deployed as a web server application. This way, users have an option to keep privacy of their datasets without sending over their data through internet to the server provided by the software owner. Also, users may analyze their data using most updated information by a single click that updates the data source files (e.g. GO files). *GOAL *presents its result of analysis in a highly visual and interactive manner. The unique automated analysis capabilities of *GOAL *combined with its visualization and integration capabilities should merit *GOAL *to be the software tool of choice for functional evaluation of gene groups. *GOAL *is available in Additional File 1. Subsequent update will be available at *GOAL *home page: http://bioinfo.iit.nrc.ca/GOAL/.

## Availability And Requirements

**Software name**: GOAL: **G**ene **O**ntology **A**na**L**yzer

**Project home page**: http://bioinfo.iit.nrc.ca/GOAL/

**Operating system(s)**: Tested in Microsoft Windows (XP, Vista) and Linux (Ubuntu 9.10); technically, it should work with all operating systems supporting Java 1.6 or higher.

**Programming language**: Java

**Other requirements**: Java 1.6 or higher, internet connection

**License**: non-commercial research use license

**Any restrictions to use by non-academics**: license needed from authors for commercial use.

## Authors' contributions

ABT initiated the *GOAL *project. ABT and YP both contributed to the design of *GOAL*. AG implemented *GOAL*. All participated in the drafting and revising of the manuscript.

## Supplementary Material

Additional file 1 *GOAL *jar file and *GOAL *user manual (*GOAL*-1.0.zip). Click here for file

Additional file 2 Quick video tutorial of GOAL (GOAL_Quick_Video_Tutorial.zip) (HTML, JavaScript & Shockwave Flash files). Require web browser to visualize. This tutorial is also accessible via the GOAL project website at the following URL: http://bioinfo.iit.nrc.ca/GOAL/demo/GOAL-Demo.htm Click here for file

Additional file 3 *GOAL *(GO biological process and KEGG pathway association analysis) and GOSt [19] detail results of six human genes: IFNA4 IL12B IL2RB STAT1 STAT2 IRF9. They were provided to us by one of the reviewers. Click here for file
