# Supplementary material for: GOAL: A software tool for assessing biological significance of genes groups
Source: BMC Bioinformatics. 2010 May 6;11:229. doi: 10.1186/1471-2105-11-229 (PMC2873542; doi:10.1186/1471-2105-11-229)
Supplement: Additional file 1 — GOAL jar file and GOAL user manual (GOAL-1.0.zip). [file 1471-2105-11-229-S1.ZIP › GOAL-1.0/Documentation/GOAL_manual.pdf]

GOAL: Gene Ontology Analyzer Manual

Bioinformatics Team
Institute for Information Technology
National Research Council Canada
1200 Montreal Road, Ottawa, Ontario, Canada K1A 0R6

1 INTRODUCTION **3**

2 MANUAL OVERVIEW **3**

3 PRELIMINARIES **3**

3.1 SYSTEM REQUIREMENTS 3
3.2 INSTALLATION 3
3.3 CONFIGURING *GOAL* 3
3.4 CONFIGURING *GOAL* SERVER/CLIENT 4
3.4.1 *GOAL* Client Setup: 4
3.4.2 *GOAL* RMI Server Setup: 4
3.4.3 *GOAL* Client Web Start (Advanced): 4

4 RUNNING *GOAL* **5**

4.1 USING GUI: INPUT 5
4.2 OUTPUT 7
4.3 COMMAND LINE INTERFACE (CLI) 8

5 RUNNING *GOAL* WEB SERVER **9**

5.1 STARTING *GOAL* SERVER 9
5.2 *GOAL* CLIENT SETUP 10

6 UNDERSTANDING *GOAL* INPUT AND OUTPUT **10**

6.1 INPUT FILE FORMATS 10
6.2 OUTPUT FILE FORMATS 11

7 DEVELOPER INFORMATION (ADVANCED) **11**

7.1 USE AS PLUG-IN 11
7.2 ADDING AND RETRIEVING SPECIES 12
7.3 FILE SYSTEM 12

1 Introduction

GOAL is an application that groups genes based on their relationships defined in Gene Ontology (GO: http://www.geneontology.org/), transcription factors (TF) that co-regulate them and their association with KEGG pathways (http://www.genome.jp/kegg/pathway.html). GO relationships are derived by analyzing annotation and ontology (obo format) files. Once the genes are grouped by their association with GO, TF or KEGG pathway, they are analyzed for statistical significance using p-values. The results interface then displays the sorted data and provides links to the gene ontology website. A video tutorial for the first time users is available at http://bioinfo.iit.nrc.ca/GOAL/tutorials.html.

2 Manual Overview

This manual covers all one needs to know to use *GOAL* successfully. First it covers installation and configuration, then describes how to run *GOAL*, and finally includes a section for developers.

3 Preliminaries

3.1 System Requirements

GOAL is implemented entirely in Java. It is available as an executable jar file and works with Java SDK 1.6 or later (http://www.java.com/en/download/index.jsp) under Windows and Linux operating system. The java virtual machine must be set properly before running *GOAL*. This is done automatically with the runGOAL.bat or runGOAL_linux file before they run *GOAL*. Specifically the commands are:

java
*-Xms32m –Xmx*1064m
-Djava.security.policy=server.policy (server)
-Djava.security.policy=client.policy (client)

3.2 Installation

Simply extract the content of the zip file into the desired install location. The executable JAR and supporting files will be located in the "*GOAL*" folder after extraction.

3.3 Configuring *GOAL*

A configuration file with default values is generated at runtime. If the user wishes to use non-default directories/download locations or wishes to run *GOAL* as a web server, changes may be made in the file. In most cases, no change in configuration is needed.

GOAL uses a simple config.prop file to store directories and URLs. If this file does not exist it is created with default values. This file is in the standard java property file format,

with keys on the left and the values on the right. The first two lines are for the annotation files and the ontology file. The next four lines are for the download URL and additional URL tags for downloading annotation/ontology files. The URL tags are needed after the file name for certain download locations. The next line is for the local IP address if *GOAL* is to be used as a server. The final line is for synonym files if they exist.

```
#Fri Oct 02 14:50:51 EDT 2009
Annotation_Dir=D\:\\GOAnalyzer\\Deployment\\Data\\gofiles\\
Ontology_File=D\:\\GOAnalyzer\\Deployment\\Data\\gofiles\\gene_ontology_edit.obo
Annotation_URL=http\://www.geneontology.org/gene-associations/
Annotation_Ext=
Ontology_URL=http\://www.geneontology.org/ontology/
Ontology_Ext=
Server_IP=10.10.23.145
Synonym_Dir=D\:\\GOAnalyzer\\Deployment\\Data\\goSynonyms\\
```

Figure 1: Example configuration.

3.4 Configuring *GOAL* Server/Client

3.4.1 *GOAL* Client Setup:

The *GOAL* client is a special mode of *GOAL* where only GUI components are in use. All analysis is done on a *GOAL* installation running the *GOAL* server. Since it is only an interface for the server no configuration is needed if Java is correctly installed. For even simpler use for the end user, see section 3.4.3 *GOAL* Client Web Start.

3.4.2 *GOAL* RMI Server Setup:

The *GOAL* server requires no configuration unless it is being started from command line (“runServer.bat”). The port value is set to the default port 1099 and will be acceptable if the port is not in use. If it is in use, modify runGOALCLI.bat/runServer.bat port entry: --port=<available port> .

3.4.3 *GOAL* Client Web Start (Advanced):

Before deploying an application with Java Web Start over the Web, the user must ensure that the Web server is able to handle JNLP files. Configure the Web server so that files with the *.jnlp extension are set to the application/x-java-jnlp-file MIME type. The way to set the JNLP MIME type depends on the respective Web server. For example, for the Apache Web server, one simply adds the line

application/x-java-jnlp-file JNLP

to the mime.types file. For other Web servers, check the documentation for instructions on setting MIME types.

Next, copy the "*GOAL*" folder in "Deployment/WebServer" to the location that your web server stores web pages. (e.g. htdocs for Apache). Modify the jnlp file, under the "codebase" tag to match the URL of the web server. This should allow users to web start the client GUI. Alternatively one can distribute the *GOAL* installer to users on other machines to manually install clients.

4 Running *GOAL*

4.1 Using GUI: Input

To run *GOAL* double click on run*GOAL*-windowns.bat or run*GOAL*-linux. **Figure 2** shows *GOAL*'s Graphical User Interface (GUI). It has settings for file input and output, algorithm parameters, a run button, and a progress indicator.

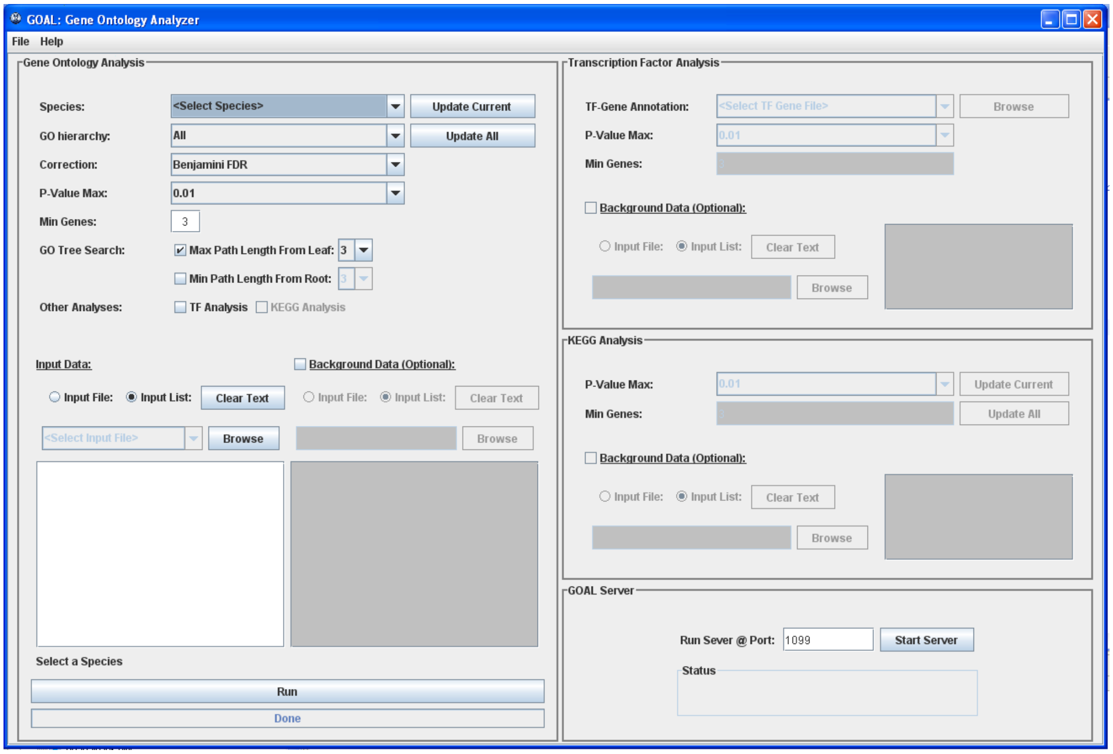

Figure 2: *GOAL* GUI using Java's AWT and Swing

When the GUI starts up, it first runs some preprocessing in the background. During this time you cannot run *GOAL*. However this should only take at most a few seconds and does not disable parameter entry.

The first step is to enter the parameters. At startup all parameters are set to default. The only essential parameter is the species, since no results will be display if it does not match the species of the input genes. Beside the species combo box there are two buttons labeled "Update All" and "Update Current". If these buttons are clicked they will download the latest GO annotations for

all species or the currently selected species, respectively. The former will also download the ontology file. **Table 1** summarizes the input parameters.

Table 1: Overview of input parameters.

Parameter	Description
`Species`	Species of the input genes. This is required to determine the correct annotation file.
`GO hierarchy`	Allows a choice of either the whole ontology or a specific GO category: biological process, cellular component or molecular function.
`Correction`	Multiple testing correction type for correcting p-values. The choices are Bonferroni, Bonferroni step-down and Benjamini False Discovery Rate listed in order of decreasing stringency. For example, Bonferroni will have fewest false positives at the cost of increased false negatives.
`P-value`	This parameter sets the p-value filter on the results. Only p-values equal to or better than the specified value will be shown.
`Min Genes`	This parameter sets a filter for the minimum number of gene in the results. Only groupings with equal to or more than the specified number of genes will be shown.
`GO tree search`	
`Max path Length From Leaf`	Maximum number of steps of parental relationships considered during grouping. Genes are matched to their associated GO term and then trace the parental path to related GO terms. The lower this value is the more specific the GO terms are.
`Min Path Length From Root`	Minimum number of GO paths from the root (e.g. Biological Process) that a GO term must be for it to be included in the analysis. The higher this value is the more specific the GO terms are.

The *Max Path Length From Leaf* and *Min Path Length From Root* are check boxes. This is because they could be used separately or together which defines the algorithm used. At least one must be checked.

Below the parameters there are check boxes to select other analyses, transcription factor and KEGG analyses. This is an additional function that runs after GO analysis that groups genes by their associated transcription factors (TF) or KEGG pathways based on a provided annotation (see 6.1 Input File Formats). By checking either or both check boxes, the corresponding panels to the right of the main panel become active. Both *Transcription Factor Analysis* and *KEGG Analysis* panels allow for the selection of thresholds for p-values and minimum number of genes in each group. Users may also choose to enter a background list of genes specific for the analysis by clicking the check box at the bottom of each panel. In addition, the TF analysis panel has a drop down list of available TF annotation files corresponding to different species and also allows for the user to add their own to the list via the “Browse” button. KEGG analysis can only be

activated if a supported species is selected. Supported species includes ***Saccharomyces cerevisiae, Arabidopsis thaliana, Homo sapiens, Mus musculus,*** **and *Rattus norvegicus.***

The next section is split into two parts, on the left is a section provided for inputting gene list. The right section is optional, enabled by a check box, for specifying background data. Both these sections allow the user to choose between specifying an input file or entering/pasting a list of genes. The background data is used for p-value calculations and could improve accuracy for specific situations. For example, if a user is analyzing a cluster derived from a microarray experiment, the probe set (gene IDs) printed on the microarray could make a better background than the entire genome, which is used by default. The default type of gene ID accepted is the default for the database for the selected species and given under the textfield for gene IDs. Key species (*Arabidopsis*, Yeast, Human, etc.) have greater gene ID support that allows most common types of gene IDs. When such species is selected, "various IDs accepted" shows under the textfield. The input file format is specified in section 6.1 Input File Formats. Sample input for *Saccharomyces cerevisiae* is provided.

The last panel on the bottom right is to enable the *GOAL* RMI Server. This is an optional field and should only be used if there are clients that wish to use the current installation as a server. Here a free port (1024-49151) must be specified. *GOAL* will display an error message if the port is in use.

After all this information is entered, press the large "Run" button on the bottom left to start running the analysis.

4.2 Output

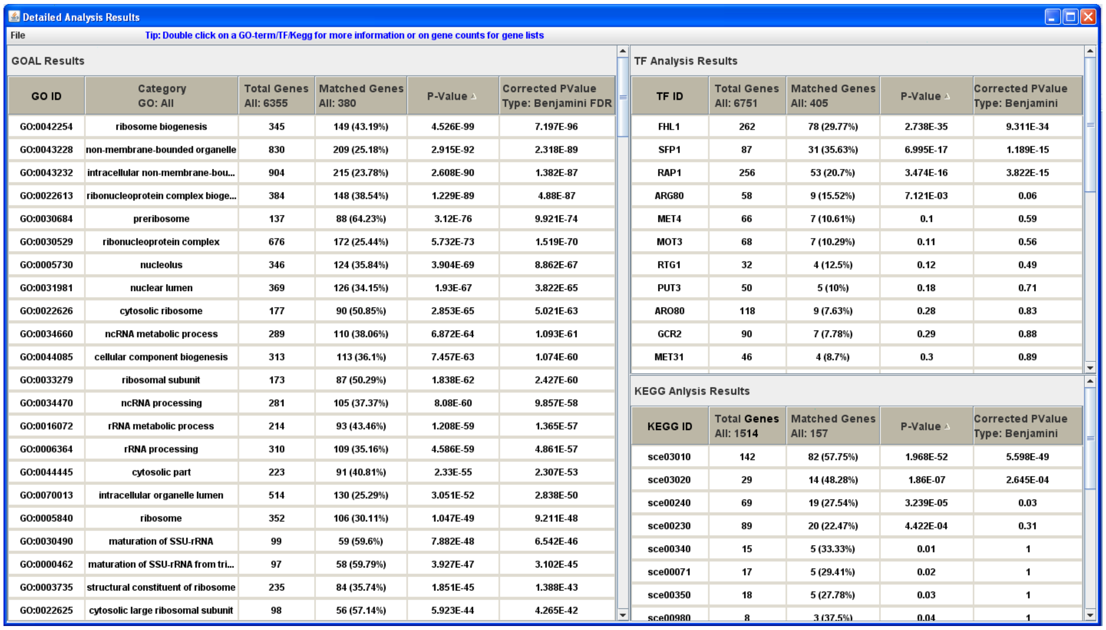

Detailed Analysis Results

File   Tip: Double click on a GO-term/TF/Kegg for more information or on gene counts for gene lists

GOAL Results

GO ID	Category GO: All	Total Genes All: 6355	Matched Genes All: 380	P-Value	Corrected PValue Type: Benjamini FDR
GO:0042254	ribosome biogenesis	345	149 (43.19%)	4.526E-99	7.197E-96
GO:0043228	non-membrane-bounded organelle	830	209 (25.18%)	2.915E-92	2.318E-89
GO:0043232	intracellular non-membrane-bou...	904	215 (23.78%)	2.608E-90	1.382E-87
GO:0022613	ribonucleoprotein complex bioge...	384	148 (38.54%)	1.229E-89	4.88E-87
GO:0030684	preribosome	137	88 (64.23%)	3.12E-76	9.921E-74
GO:0030529	ribonucleoprotein complex	676	172 (25.44%)	5.732E-73	1.519E-70
GO:0005730	nucleolus	346	124 (35.84%)	3.904E-69	8.862E-67
GO:0031981	nuclear lumen	369	126 (34.15%)	1.93E-67	3.822E-65
GO:0022626	cytosolic ribosome	177	90 (50.85%)	2.853E-65	5.021E-63
GO:0034660	ncRNA metabolic process	289	110 (38.06%)	6.872E-64	1.093E-61
GO:0044085	cellular component biogenesis	313	113 (36.1%)	7.457E-63	1.074E-60
GO:0033279	ribosomal subunit	173	87 (50.29%)	1.838E-62	2.427E-60
GO:0034470	ncRNA processing	281	105 (37.37%)	8.08E-60	9.857E-58
GO:0016072	rRNA metabolic process	214	93 (43.46%)	1.208E-59	1.365E-57
GO:0006364	rRNA processing	310	109 (35.16%)	4.586E-59	4.861E-57
GO:0044445	cytosolic part	223	91 (40.81%)	2.33E-55	2.307E-53
GO:0070013	intracellular organelle lumen	514	130 (25.29%)	3.051E-52	2.838E-50
GO:0005840	ribosome	352	106 (30.11%)	1.047E-49	9.211E-48
GO:0030490	maturation of SSU-rRNA	99	59 (59.6%)	7.882E-48	6.542E-46
GO:0000462	maturation of SSU-rRNA from tri...	97	58 (59.79%)	3.927E-47	3.102E-45
GO:0003735	structural constituent of ribosome	235	84 (35.74%)	1.851E-45	1.388E-43
GO:0022625	cytosolic large ribosomal subunit	98	56 (57.14%)	5.923E-44	4.265E-42

TF Analysis Results

TF ID	Total Genes All: 6751	Matched Genes All: 405	P-Value	Corrected PValue Type: Benjamini
FHL1	262	78 (29.77%)	2.738E-35	9.311E-34
SFP1	87	31 (35.63%)	6.995E-17	1.189E-15
RAP1	256	53 (20.7%)	3.474E-16	3.822E-15
ARG80	58	9 (15.52%)	7.121E-03	0.06
MET4	66	7 (10.61%)	0.1	0.59
MOT3	68	7 (10.29%)	0.11	0.56
RTG1	32	4 (12.5%)	0.12	0.49
PUT3	50	5 (10%)	0.18	0.71
ARO80	118	9 (7.63%)	0.28	0.83
GCR2	90	7 (7.78%)	0.29	0.88
MET31	46	4 (8.7%)	0.3	0.89

KEGG Anlysis Results

KEGG ID	Total Genes All: 1514	Matched Genes All: 157	P-Value	Corrected PValue Type: Benjamini
sce03010	142	82 (57.75%)	1.968E-52	5.598E-49
sce03020	29	14 (48.28%)	1.86E-07	2.645E-04
sce00240	69	19 (27.54%)	3.239E-05	0.03
sce00230	89	20 (22.47%)	4.422E-04	0.31
sce00340	15	5 (33.33%)	0.01	1
sce00071	17	5 (29.41%)	0.02	1
sce00350	18	5 (27.78%)	0.03	1
sce00980	8	3 (37.5%)	0.04	1

Figure 3: *GOAL* after execution.

When *GOAL* finishes running, the output dialogs appear (**Figure 3**); otherwise, an error message is displayed in the progress bar if the system encounters an error. If no error occurred,

the result windows will open. If any genes in the query gene list were not found from the annotation file, the IDs of these genes are displayed in separate windows, one for each analysis (GO, TF-gene association, or KEGG association).

Next, it displays up to three result windows, each containing a table. The tables are sortable and can be copied to clipboard. The first column is the group identifier (GO/TF/KEGG ID) with a second column for a description in the case of GO ID. If this column is double clicked, a browser will open and display the Amigo (http://amigo.geneontology.org/cgi-bin/amigo/go.cgi), DBD (http://dbd.mrc-lmb.cam.ac.uk/DBD/index.cgi) or KEGG (e.g. http://www.genome.jp/dbget-bin/www_bget?hsa00970) entry for the GO/TF/KEGG ID. The next two columns show the number of genes matched to the group from the background data and the input data respectively. If these are double clicked the actual gene ids will be displayed in another dialog along with their associated KEGG IDs (if available), which can be saved to a file (see 6.2 Output File Formats for details). These cells also open a browser to display additional information. The final two columns include the p-value and corrected p-value of each group.

A menu bar is also included in the above tables. They allow users to save the data to a file or close the window. Any number of windows can be open at a time so multiple results can be compared.

4.3 Command Line Interface (CLI)

Figure 4 shows a Command Line Interface (CLI) version of the GUI shown in **Figure 3**. Users may prefer this when running *GOAL* as part of a tool suite. Default values are made available through run*GOAL*-windows.bat or run*GOAL*_linux. Either --gui, --client, --server or *GOAL* parameters must be included. If the *GOAL* parameters are used all input and output options must be used as well (except for optional TF-gene/KEGG). --gui and --server ignore other options. --client may be combined with –gui to display the client GUI. Currently one parameter, degree, is used for both *Max Path Length From Leaf* and *Min Path Length From Root* seen in the GUI. Also p-values, minGenes and background are equal for all analyses.

```
Startup:
  -h,  --help
  -v,  --version

IO:
  -i,  --input-file=FILE path to gene names data file
  -t,  --tf-file=FILE path to TF-gene annotation (Optional)
  -k,  --kegg-file=FILE path to KEGG annotation (Optional)
  -b,  --background-file=FILE path to background gene names data
         file (Optional, annotation will be used if null)
  -o,  --output-dir=DIR  path to output results

UI:
       --gui   run with gui. Implies necessary fields

GOAL:
       --species=STR     Name of species (Must have annotation)
       --hierarchy=STR
         biological_process/molecular_function/cellular_component
       --correction=STR  Benjamini/Bonferroni/BonferroniSD
       --maxParental=INT Max path length from leaf
        --minRoot=INT    Min path length from root
       --pvalue=DOUBLE   Max P value
       --minGenes=INT    Min genes required in a group

Client/Server:
       --client          run client. Implies necessary fields
       --server          Run server. Port required
  -s,  --serverIp=STR    IP address of started RMI server
  -p,  --port=INT        Port used for RMI server

Configuration:
       --config-file     Path to configuration file
```

Figure 4: Output of *GOAL* –help

5 Running *GOAL* Web Server

5.1 Starting *GOAL* Server

The *GOAL* Server can be started in two ways, through the GUI or through command line. Through the GUI there is an optional field that allows a port to be specified (**Figure 2**). Here a free port (1024-49151) must be specified. *GOAL* will display an error message if the port is in use. This port must match the port specified by the clients.

Through command line the jar must run with the option --server –port=<port>. A script file runServer-windows.bat or runServer-linux is provided. The window must remain open for the server to continue running. If one wishes to stop the server, type "stop" and press enter. Refer to "readme" file in the "WebServer" folder for details.

5.2 *GOAL* Client Setup

The *GOAL* client is very similar to the main *GOAL* program in terms of how it is used. The client can be launched from a normal install as well as web start support outlined in section 3.3.1. For the installed version, batch files are included to run the client just as in *GOAL*. However, small differences in both the GUI exist (**Figure 5**). The interface of the client lacks the update buttons, TF Gene browse button and the server setup panel. This is because the client does not manage any annotations or ontologies. They are all located on the server so that they only need to be managed in one place. There is no server setup panel since the client cannot be used as a server. The lack of TF-gene browse button is also due to all the TF-gene annotations being located at the server. What this interface has in addition to the server interface is a client setup panel. This is required to specify where the server is located to allow a connection to be established. The other panels will remain disabled until this information is specified.

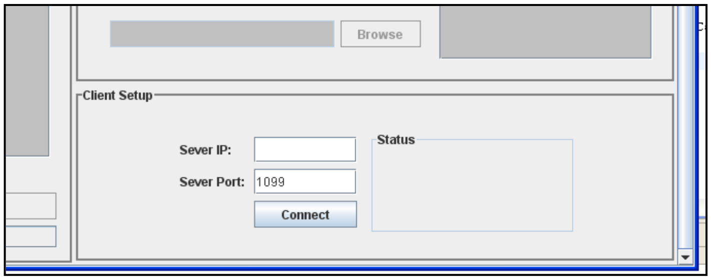

Figure 5: Client setup interface of *GOAL* Client

To run the client from command line, use the options –client –port=<port> -serverIp<IP address of server>. See section 4.3 for details.

6 Understanding *GOAL* Input and Output

6.1 Input File Formats

There is a variety of different input files, each with different formats. They can be categorized into 5 sections, gene list input, GO files, gene synonyms, TF-gene annotations and KEGG annotations.

Gene list input is the input of genes to be analyzed by *GOAL*. The format of these files can be simply a text file (*.txt, or *.dat) or CSV file (*.csv). The delimiter can be a tab, newline, space, comma, or colon character.

The GO files folder includes the Gene Ontology annotation files and ontology files (obo format). Format details are available on the Gene Ontology website at: http://www.geneontology.org/GO.contents.doc.shtml.

Gene synonyms files are simple CSV files that contain the gene in the first column, and synonyms in all other columns. There must also be a header to the file and the file must be named by the extension of the corresponding GO annotation file. For example, gene_associations.tair → tair.csv.

The TF-gene association file is compiled by the user. As we mentioned earlier, the TF-gene association data can be derived from Chromatin Immunoprecipitation (ChIP) experiments. They

can also be compiled from literature search and/or TF databases of well known and well characterized biological interactions such as TRANSFAC, JASPAR, and RegulonDB, etc. For example, TRANSFAC is a database on eukaryotic transcriptional regulation. The database contains data on transcription factors, their target genes and their experimentally validated binding motifs in genes. The TF-gene association data is represented as a matrix, where the rows correspond to genes and the columns to transcription factors. The entries of the matrix are either 0 or 1, with 1 for a known interaction between a TF and its target gene and 0 for no interaction.

The KEGG pathway file contains a list of genes and their associated KEGG pathway.

6.2 Output File Formats

Output files are all CSV files with comments in the header that provide information about the parameters used to obtain the results. The rest of the file mirrors that of the output GUI table with an additional column including all the matched genes for the group of that row. They are comma delimitated as well.

7 Developer Information (Advanced)

This section is for people who would like to use this application as a plug-in.

7.1 Use as Plug-in

GOAL was designed for easy integration into other java tools. To run *GOAL* as part of another tool first the JAR file must be included in the project build path. All the functionality of *GOAL* is made available through a class call `GOALPlugin`. The way this class can be used is very flexible. The input and output GUIs can be displayed or returned to be integrated in an existing GUI. Either way they are fully functional by using `ActionListeners` within the classes created and supplemented with listeners from the `GOALPlugin` class itself.

If the GUI is not needed then *GOAL* can be used independently through this class. This is also achieved in two ways, through a regular method call or by returning a started thread as a `GOAnalysisTask`, `TFAnalysisTask`, or `KEGGAnalysisTask`. These classes can be used like normal java threads which throw a `PropertyChangeEvent` when complete. If the user is unfamiliar with `PropertyChangeListeners` the `GOALPlugin` class can be used as a listener and will invoke the results GUI when the thread is complete. Furthermore these methods include versions that run with default parameters if the user is confused by them.

The last functionality provided by this class is the ability to run the *GOAL* server. This way the server can run in the background without the GUI.

If the user does not want to deal with coding at all then *GOAL* can be used through command line with full functionality, with or without GUI.

7.2 Adding and Retrieving Species

As more annotation files are created they may need to be added to *GOAL*. This is done simple by adding a line to the constructor of SpeciesExtMap. The key is the species and the value is the extension of the annotation file.

e.g. m_mapping.put("Drosophila melanogaster", "fb");

The function getSpecieNames() handles the retrieving of the species names. The Vector<String> returned can be used to populate whatever input GUI that is used.

7.3 File System

When *GOAL* is installed a file system is created and must remain intact for *GOAL* to function properly (unless the configuration file is modified). Any new files must be in their assigned locations and named correctly.

Table 2: File System

Package	Description
Root (*GOAL*-<version>)	Main Jar, batch files, config.txt
.Deployment.data.input	Input files
.Deployment.data.gofiles	Annotations and Ontology files
.Deployment.data.goSynonyms	Synonym files (corresponding to annotation extensions)
.Deployment.data.tfgeneFiles	TF-gene annotations, named by specie
.Deployment.data.keggFiles	KEGG annotations
.Deployment.Images	Images
.Deployment.WebServer.*GOAL*	Web Start folder
.Deployment.WebServer	Server setup instructions, server.policy, batch to run server
.Documentation	System document and manual
