# Supplementary material for: GOAL: A software tool for assessing biological significance of genes groups
Source: BMC Bioinformatics. 2010 May 6;11:229. doi: 10.1186/1471-2105-11-229 (PMC2873542; doi:10.1186/1471-2105-11-229)
Supplement: Additional file 1 — GOAL jar file and GOAL user manual (GOAL-1.0.zip). [file 1471-2105-11-229-S1.ZIP › GOAL-1.0/Documentation/GOAL_tutorial.pdf]

GOAL: Gene Ontology Analyzer Tutorial

Bioinformatics Team
Institute for Information Technology
National Research Council Canada
1200 Montreal Road, Ottawa, Ontario, Canada K1A 0R6

1 Introduction

GOAL is an application that groups genes based on their relationships defined in Gene Ontology (GO: http://www.geneontology.org/), transcription factors (TF) that co-regulate them and their association with KEGG pathways (http://www.genome.jp/kegg/pathway.html). GO relationships are derived by analyzing annotation and ontology (obo format) files. Once the genes are grouped by their association with GO, TF or KEGG pathway, they are analyzed for statistical significance using p-values. The results interface then displays the sorted data and provides links to the gene ontology website. A video tutorial for the first time users is available at http://bioinfo.iit.nrc.ca/GOAL/tutorials.html.

2 Overview

This tutorial covers all one needs to know to use *GOAL* successfully. First it covers installation and configuration then describes how to run *GOAL* step-by-step, and finally explain the input and output.

3 Preliminaries

3.1 System Requirements

GOAL is implemented entirely in Java. It is available as an executable jar file and works with Java SDK 1.6 or later (http://www.java.com/en/download/index.jsp) under Windows and Linux operating system. The java virtual machine must be set properly before running *GOAL*. This is done automatically with the runGOAL.bat or runGOAL_linux file before they run *GOAL*.

3.2 Installation

Simply extract the content of the zip file to the folder where one needs to install the GOAL.

3.3 Configuring *GOAL*

GOAL uses a simple config.prop file generated by default to store directories and URLs. If this file does not exist it is created with default values. This file is in the standard java property file format, with keys on the left and the values on the right. The first two lines are for the annotation files and the ontology file. The next four lines are for the download URL and additional URL tags for downloading annotation/ontology files. The URL tags are needed after the file name for certain download locations. The next line is for the local IP address if *GOAL* is to be used as a server. The final line is for synonym files if they exist.

```
#Fri Oct 02 14:50:51 EDT 2009
Annotation_Dir=D\:\\GOAnalyzer\\Deployment\\Data\\gofiles\\
Ontology_File=D\:\\GOAnalyzer\\Deployment\\Data\\gofiles\\gene_ontology_edit.obo
Annotation_URL=http\://www.geneontology.org/gene-associations/
Annotation_Ext=
Ontology_URL=http\://www.geneontology.org/ontology/
Ontology_Ext=
Server_IP=10.10.23.145
[illegible]
```

Figure 1: Example configuration.

4 Step-by-Step Running of *GOAL*

4.1 Using GUI: Input

To run *GOAL* double click on runGOAL-windonws.bat or runGOAL-linux. **Figure 2** shows *GOAL*'s Graphical User Interface (GUI). It has settings for file input and output, algorithm parameters, a run button, and a progress indicator. When the GUI starts up, it first runs some preprocessing in the background. This should only take at most a few seconds and does not disable parameter entry.

Step 1 - Parameters:

The first step is to enter the parameters. At startup all parameters are set to default. The only essential parameter is the species; no results will be display if it is incorrect. Beside the species combo box there are two buttons labeled “Update All” and “Update Current”. If these buttons are clicked they will download the latest annotations for all species or the currently selected species respectively. The former will also download the ontology file. **Table 1** summarizes the input parameters.

Table 1: Overview of input parameters.

Parameter	Description
`Species`	Species of the input genes. This is required to determine the correct annotation file.
`GO hierarchy`	Allows a choice of either the whole ontology or a specific GO category: biological process, cellular component or molecular function.

Correction	Multiple testing correction type for correcting p-values. The choices are Bonferroni, Bonferroni step-down and Benjamini False Discovery Rate listed in the order of decreasing stringency. For example, choosing Bonferroni will have fewest false positives at the cost of increased false negatives.
P-value	This parameter sets the p-value filter on the results. Only p-values equal to or better than the specified value will be shown.
Min Genes	This parameter sets a filter for the minimum number of gene in the results. Only groupings with equal to or more than the specified number of genes will be shown.
GO tree search	
Max path Length From Leaf	Maximum number of steps of parental relationships considered during grouping. Genes are matched to their associated GO term and then trace the parental path to related GO terms. The lower this value is the more specific the GO terms are.
Min Path Length From Root	Minimum number of GO paths from the root (e.g. Biological Process) that a GO term must be for it to be included in the analysis. The higher this value is the more specific the GO terms are.

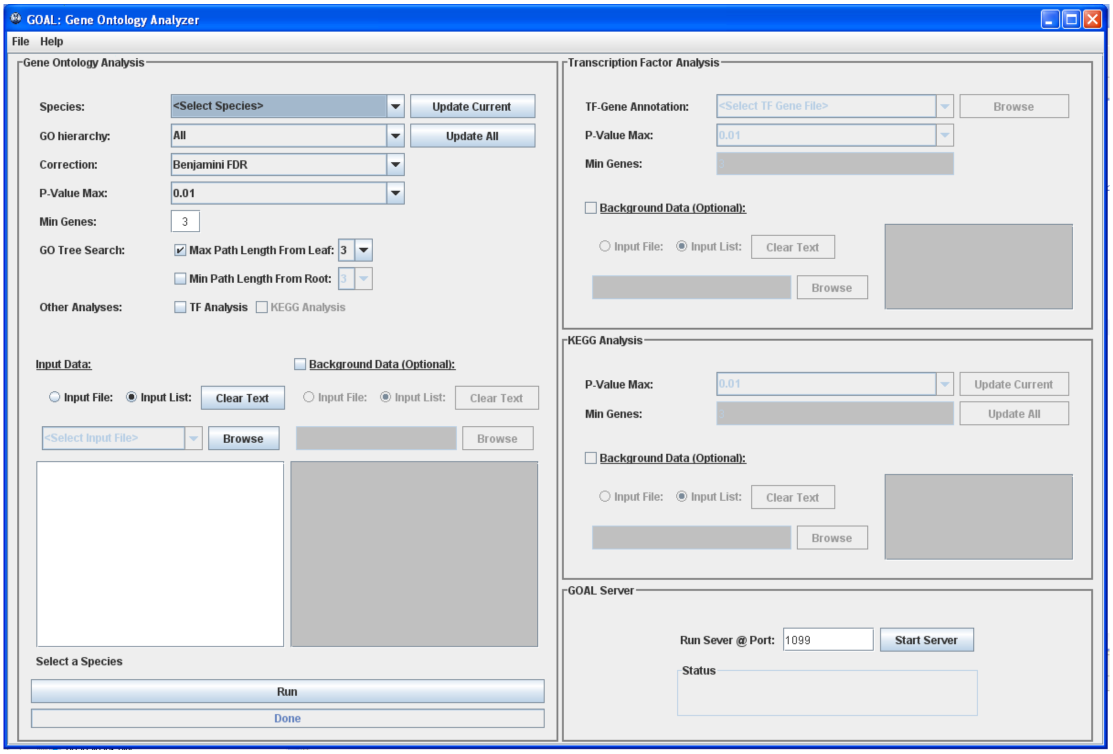

Figure 2: GOAL GUI using Java's AWT and Swing

Step 2 – Algorithm Type:

The next step is to select the algorithm type. There are two options, *Max Path Length From Leaf* and *Min Path Length From Root*, which are check boxes in the GUI. They could be used separately or together as a combined algorithm. At least one must be checked.

Step 3 – Other Analyses:

Below the parameters there are check boxes to select other analyses, transcription factor and KEGG analyses. This is an additional function that runs after GO analysis that groups genes by their associated transcription factor (TF) and/or KEGG pathway based on a provided annotation (see 6.1 Input File Formats). By checking either check box the panels to the right of the main panel become active. Both panels allow for the selection of p-values, minimum genes and a background list specific for the analysis. The TF panel also has a drop down list of available TF annotation files and also allows for the user to add their own to the list via the "Browse" button. KEGG analysis can only be activated if a supported species is selected. Supported species includes ***Saccharomyces cerevisiae, Arabidopsis thaliana, Homo sapiens, Mus musculus,*** **and** ***Rattus norvegicus.***

Step 4 – Input:

The next section is split into two parts, on the left is a section provided for inputting gene list. The right section is optional, enabled by a check box, for specifying background data. Both these sections allow users to choose between specifying an input file or entering/pasting a list of genes. The background data is used for p-value calculations and could improve accuracy for specific situations. For example, if a user is analyzing a cluster derived from a microarray experiment, the probe set (gene IDs) printed on the microarray could make a better background than the entire genome, which is used by default. <u>The default type of gene ID accepted is the default for the database for the selected species and given under the textfield for gene IDs.</u> Key species (*Arabidopsis*, Yeast, Human, etc.) have greater gene ID support that allows most common types of gene IDs. When such species is selected, "various IDs accepted" shows under the textfield. The input file format is specified in section 6.1 Input File Formats. Sample input for *Saccharomyces cerevisiae* is provided.

Step 5 – Server (optional):

The last panel on the bottom right is to enable the *GOAL* RMI Server. This is an optional field and should only be used if there are clients that wish to use the current installation as a server. Here a free port (1024-49151) must be specified. *GOAL* will display an error message if a port is in use.

Step 6 – Run:

After all this information is entered, press the large "Run" button to start running the analysis.

4.2 Using GUI: Output

When *GOAL* finishes running, the output dialogs appear (**Figure 3**); otherwise, an error message is displayed in the progress bar if the system encounters an error. If no error occurred, the result windows will open. If any genes in the query gene list were not found from the annotation file, the IDs of these genes are displayed in separate windows, one for each analysis (GO, TF-gene association, or KEGG association).

Next, it displays up to three result windows, each containing a table. The tables are sortable and can be copied to clipboard. The first column is the group identifier (GO/TF/KEGG ID) with a second column for a description in the case of GO ID. If this column is double clicked, a browser will open and display the Amigo (http://amigo.geneontology.org/cgi-bin/amigo/go.cgi), DBD (http://dbd.mrc-lmb.cam.ac.uk/DBD/index.cgi) or KEGG (e.g. http://www.genome.jp/dbget-bin/www_bget?hsa00970) entry for the GO/TF/KEGG ID. The next two columns show the number of genes matched to the group from the background data and the input data respectively. If these are double clicked the actual gene ids will be displayed in another dialog along with their associated KEGG IDs (if available), which can be saved to a file (see 6.2 Output File Formats for details). These cells also open a browser to display additional information. The final two columns include the p-value and corrected p-value of each group.

A menu bar is also included in the above tables. They allow users to save the data to a file or close the window. Any number of windows can be open at a time so multiple results can be compared.

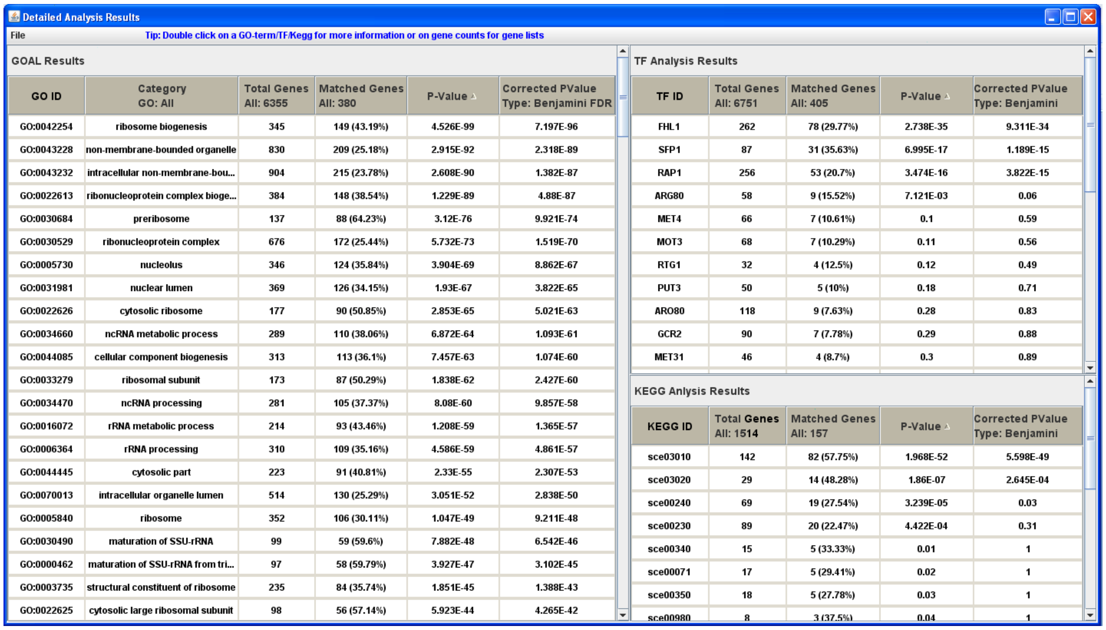

Figure 3: *GOAL* after execution.

5 Understanding Input and Output

5.1 Input File Formats

There are a variety of different input files, each with different formats. They can be categorized into 5 sections, gene list input, GO files, gene synonyms, TF-gene annotations, and KEGG associations.

Gene list input is the input of genes to be analyzed by *GOAL*. The format of these files can be simply a text file (*.txt, or *.dat) or CSV file (*.csv). The delimiter can be a tab, newline, space, comma, or colon character.

Details of other files are provided with the deployment and their details are available in the User Manual.

5.2 Output File Formats

Output files are all CSV files with comments in the header that provide information about the parameters used to obtain the results. The rest of the file mirrors that of the output GUI table with an additional column including all the matched genes for the group of that row. They are comma delimitated as well. A CSV file can be opened by any spreadsheet program such as Excel in Windows.
