# Supplementary figures and images for: GOAL: A software tool for assessing biological significance of genes groups
Source: BMC Bioinformatics. 2010 May 6;11:229. doi: 10.1186/1471-2105-11-229 (PMC2873542; doi:10.1186/1471-2105-11-229)

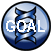

Supplement: Additional file 1 — GOAL jar file and GOAL user manual (GOAL-1.0.zip). [file 1471-2105-11-229-S1.ZIP › GOAL-1.0/Deployment/Images/goanalyzer.PNG]
