# Supplementary material for: GOAL: A software tool for assessing biological significance of genes groups
Source: BMC Bioinformatics. 2010 May 6;11:229. doi: 10.1186/1471-2105-11-229 (PMC2873542; doi:10.1186/1471-2105-11-229)
Supplement: Additional file 3 — GOAL (GO biological process and KEGG pathway association analysis) and GOSt [19] detail results of six human genes: IFNA4 IL12B IL2RB STAT1 STAT2 IRF9. They were provided to us by one of the reviewers. [file 1471-2105-11-229-S3.PDF]

Additional file 3

The GOAL analysis (GO biological process and KEGG pathways) of six human genes (IFNA4 IL12B IL2RB STAT1 STAT2 IRF9). (a) Input interface with input parameters. (b) Output interface describing the results. As can be seen, the GO biological process is consistent with the KEGG analysis. (c) GOST results of the above six genes. As can be seen, GOAL and GOST results are consistent.

GOAL input

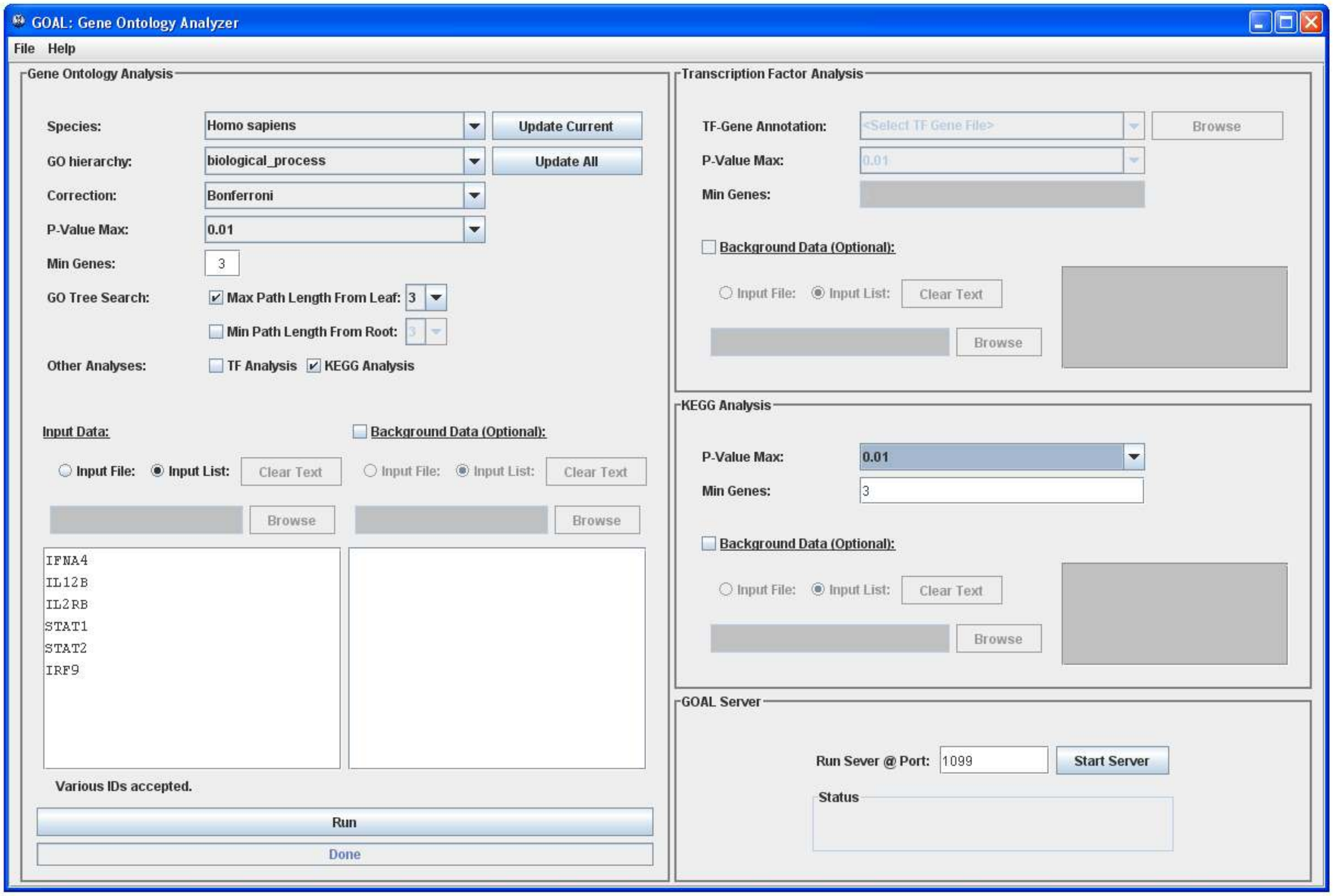

GOAL results

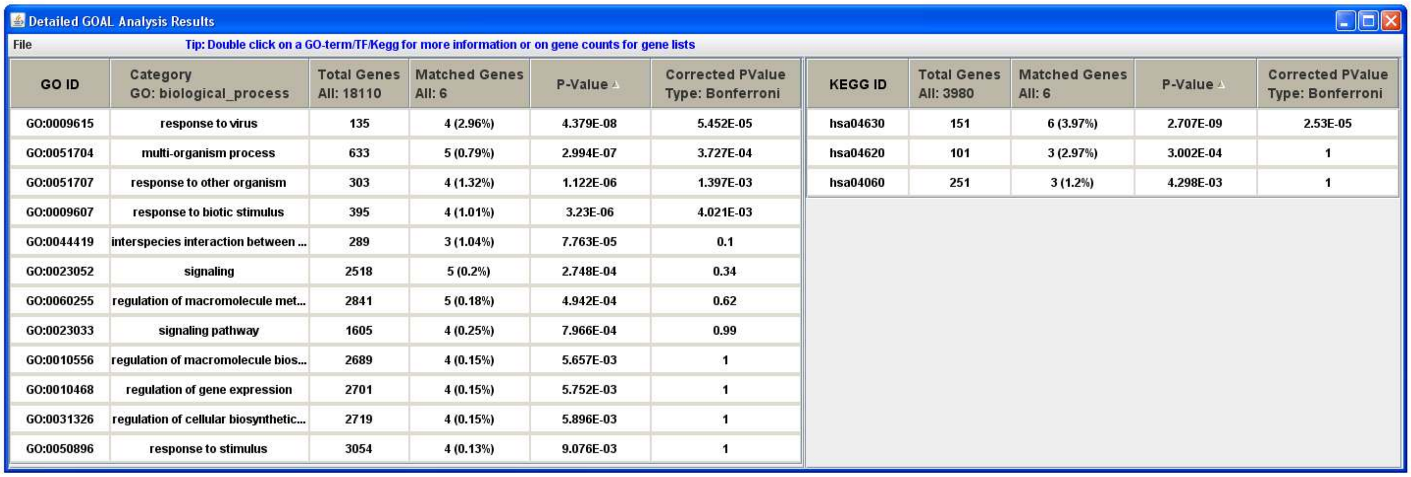

Detailed GOAL Analysis Results

File

Tip: Double click on a GO-term/TF/Kegg for more information or on gene counts for gene lists

GO ID	Category GO: biological_process	Total Genes All: 18110	Matched Genes All: 6	P-Value	Corrected PValue Type: Bonferroni
GO:0009615	response to virus	135	4 (2.96%)	4.379E-08	5.452E-05
GO:0051704	multi-organism process	633	5 (0.79%)	2.994E-07	3.727E-04
GO:0051707	response to other organism	303	4 (1.32%)	1.122E-06	1.397E-03
GO:0009607	response to biotic stimulus	395	4 (1.01%)	3.23E-06	4.021E-03
GO:0044419	interspecies interaction between ...	289	3 (1.04%)	7.763E-05	0.1
GO:0023052	signaling	2518	5 (0.2%)	2.748E-04	0.34
GO:0060255	regulation of macromolecule met...	2841	5 (0.18%)	4.942E-04	0.62
GO:0023033	signaling pathway	1605	4 (0.25%)	7.966E-04	0.99
GO:0010556	regulation of macromolecule bios...	2689	4 (0.15%)	5.657E-03	1
GO:0010468	regulation of gene expression	2701	4 (0.15%)	5.752E-03	1
GO:0031326	regulation of cellular biosynthetic...	2719	4 (0.15%)	5.896E-03	1
GO:0050896	response to stimulus	3054	4 (0.13%)	9.076E-03	1

KEGG ID	Total Genes All: 3980	Matched Genes All: 6	P-Value	Corrected PValue Type: Bonferroni
hsa04630	151	6 (3.97%)	2.707E-09	2.53E-05
hsa04620	101	3 (2.97%)	3.002E-04	1
hsa04060	251	3 (1.2%)	4.298E-03	1

GOST results

IFNA4	L12B	L2RB	STAT1	STAT2	IRF9	P-value	T	Q	Q&T	Q&T/Q	Q&T/T	term ID	term domain	and name
A		e	e	e	e	5.00e-07	753	6	5	0.833	0.007	GO:0051704	BP	multi-organism process (1)
A			e	e	e	4.83e-06	469	6	4	0.667	0.009	GO:0009607	BP	response to biotic stimulus (1)
A			e	e	e	8.69e-07	305	6	4	0.667	0.013	GO:0051707	BP	response to other organism (2)
A			e	e	e	1.61e-08	113	6	4	0.667	0.035	GO:0009615	BP	response to virus (3)
			e	A		8.53e-05	47	6	2	0.333	0.043	GO:0007259	BP	JAK-STAT cascade (1)
						P-value	T	Q	Q&T	Q&T/Q	Q&T/T	term ID	term domain	and name
			A	A		4.76e-07	4	6	2	0.333	0.500	GO:0005062	MF	hematopoietin/interferon-class (D200-domain) cytokine receptor signal trans... (1)
						P-value	T	Q	Q&T	Q&T/Q	Q&T/T	term ID	term domain	and name
k	k		k			1.53e-04	107	6	3	0.500	0.028	KEGG:04620	ke	Toll-like receptor signaling pathway (1)
k	k	k	k	k	k	5.66e-10	155	6	6	1.000	0.039	KEGG:04630	ke	Jak-STAT signaling pathway (1)

INFO PARAMETERS: sort_by_structure = 1
INFO PARAMETERS: user_thr = 1.00
INFO PARAMETERS: significant = 1
INFO PARAMETERS: organism = hsapiens
INFO PARAMETERS: analytical = 1
INFO User: g:Profiler web interface
INFO Host: ronk1.at.mt.ut.ee
INFO Time: 2010-3-2 14:51:39

IFNA4	IFNA4	Interferon alpha-4 Precursor (Interferon alpha-4B)(Interferon alpha-M1)(Interferon alpha-76) [Source:UniProtKB/Swiss-Prot;Acc:...
IL12B	IL12B	Interleukin-12 subunit beta Precursor (IL-12B)(IL-12 subunit p40)(Cytotoxic lymphocyte maturation factor 40 kDa subunit)(CLMF ...
IL2RB	IL2RB	Interleukin-2 receptor subunit beta Precursor (High affinity IL-2 receptor subunit beta)(IL-2 receptor)(P70-75)(p75)(CD122 ant...
STAT1	STAT1	Signal transducer and activator of transcription 1-alpha/beta (Transcription factor ISGF-3 components p91/p84) [Source:UniProt...
STAT2	STAT2	Signal transducer and activator of transcription 2 (p113) [Source:UniProtKB/Swiss-Prot;Acc:P52630]
IRF9	IRF9	Interferon regulatory factor 9 (IRF-9)(Transcriptional regulator ISGF3 subunit gamma)(IFN-alpha-responsive transcription facto...
